# The ecological importance of the accuracy of environmental temperature measurements

**DOI:** 10.1098/rsbl.2022.0263

**Published:** 2022-08-10

**Authors:** Melissa N. Staines, David T. Booth, Jacques-Oliver Laloë, Ian R. Tibbetts, Graeme C. Hays

**Affiliations:** ^1^ School of Biological Sciences, The University of Queensland, St Lucia, Queensland 4072, Australia; ^2^ School of Life and Environmental Sciences, Deakin University, Geelong, Victoria 3216, Australia

**Keywords:** climate warming, data loggers, sea turtles, sex ratios, temperature-dependent sex determination

## Abstract

The implications of logger accuracy and precision are rarely considered prior to their application in many ecological studies. We assessed the accuracy and precision of three temperature data loggers widely used in ecological studies (Hobo®, iButton® and TinyTag®). Accuracy was highest in TinyTags (95% of readings were within 0.23°C of the true temperature) and lowest in HOBOs and iButtons (95% of were readings within 0.43°C and 0.49°C of the true temperature, respectively). The precision (standard deviation of the repeat measurements) was greatest in TinyTags (0.04°C), followed by iButtons (0.17°C) and then HOBOs (0.22°C). As a case study, we then considered how modelled estimates of sea turtle hatchling sex ratios (derived from temperature), could vary as a function of logger accuracy. For example, at 29°C when the mean sex ratio derived was 0.47 female, the sex ratio estimate from a single logger could vary between 0.40 and 0.50 for TinyTags and 0.29 and 0.56 for both HOBOs and iButtons. Our results suggest that these temperature loggers can provide reliable descriptions of sand temperature if they are not over-interpreted. Logger accuracy must be considered in future ecological studies in which temperature thresholds are important.

## 1. Introduction

Integral to many thousands of ecological studies are measurements of environmental temperature. For example, temperature (e.g. air or sea surface temperature) is often used among a suite of environmental variables to explain the distribution of organisms and the phenology of events such as flowering, breeding and migration [1–3]. Temperature also plays a key role in the physiological ecology of species, as it often determines the energetics of torpor and hibernation [4,5], patterns of activity [6,7] or dive durations for some air-breathing marine vertebrates [8–11]. Further, the impact of climate warming on ecosystems and biodiversity is one of the most pressing ecological questions in recent decades [12,13]. For many oviparous taxa, environmental temperature also plays a key role in the development of their embryos. For example, for some fishes and many reptiles, incubation temperature differentiates the sex of offspring and determines their survival (reviewed Noble *et al*. [14]). For sea turtles, rising incubation temperatures due to climate warming may lead to increasingly female-biased hatchling cohorts, termed ‘feminization’, which in extreme cases might compromise population viability [15–19].

Environmental temperature data are gathered by scientists from weather stations held in global databases such as the UK Meteorological Office Hadley Centre-University of East Anglia Climatic Research Unit (HadCRUT, http://www.metoffice.gov.uk/hadobs/hadcrut4) [20], those collated from ships, buoys and ocean moorings around the world and stored in the International Comprehensive Ocean-Atmosphere Data Set (ICOADS, https://icoads.noaa.gov/) [21] and The Met Office Hadley Centre's Sea Surface Temperature dataset (HadSST, https://www.metoffice.gov.uk/hadobs/hadsst4/) [22]. Ecologists can then use these open-source data to create models to address environmental questions about their focal taxon (e.g. [23,24]). Data from such sources can be accompanied by the deployment of *in situ* temperature loggers (electronic supplementary material, table S1). While there are strict data quality protocols for data admitted to these global databases [20,25], the accuracy (how close the ‘measured’ temperature is to the ‘true’ temperature) and precision (how close repeat measurements are to each other) of field temperature loggers is seldom explicitly considered by researchers themselves (but see [26]).

Given the importance of *in situ* temperature measurements for those ecological studies that are driven by questions regarding the ecological consequences of different climate change scenarios, here we assess the accuracy and precision of temperature loggers. Temperature logger performance may be particularly important here, because sea turtles have temperature-dependent sex determination with the hatchling sex ratio varying with small changes in temperature [27]. Hence, we explore a case study of the importance of logger performance for estimates of the sex ratio of hatchling sea turtles derived from incubation temperature data. In this way, we consider more broadly how ecologists can consider the implications of the accuracy of their temperature measurements in their work.

## 2. Methods

### (a) Assessing the accuracy and precision of temperature loggers

We assessed the accuracy and precision of three types of temperature loggers commonly used in sea turtle incubation studies: (1) iButton® data loggers (DS1922 L-F5), manufactured by Maxim Integrated, San Jose, California (USA), (2) Pendant HOBO® data loggers (MX2201), manufactured by Onset Computer Corp. Bourne, Massachusetts (USA), and (3) TinyTag® Plus 2 model data loggers (TGP-4017), manufactured by Gemini Data Loggers Ltd, West Sussex (UK) (table 1). Hereafter, these data loggers will be referred to as iButtons, HOBOs and TinyTags, respectively.

**Table 1.** Device specifications provided by the manufacturers (M) and calculated in the present study (P) for three brands of temperature data loggers. (P) Accuracy values represent 95% of temperature readings from 27°C to 33°C, and (P) precision was calculated from the mean standard deviation of repeated measurements at each temperature.

	iButton® data logger by Maxim Integrated	pendant HOBO® data logger by ONSET	TinyTag® data logger by Gemini
model number	DS1922 L-F5	MX2201	TGP-4017
mass (g)	3.30	12.75	110.00
(M) range (°C)	−40 to 85	−20 to 70	−40 to 85
(M) accuracy (°C)	± 0.5	± 0.5	± 0.5
(P) accuracy (°C)	± 0.47	± 0.43	± 0.23
(P) precision (°C)	± 0.17	± 0.22	± 0.04
(M) resolution (°C)	0.0625	0.04	0.01
battery	finite	replaceable	replaceable
durability (IP)	water resistant (IP56)	waterproof (IP68)	waterproof (IP68)

Ten iButtons, five HOBOs and four TinyTag data loggers were used in this study and each device was programmed to log temperature every 5 min. All of the data loggers were then placed into a single, sealed plastic ziplock bag (210 × 297 mm) and submerged in a laboratory heated and recirculating water bath (Thermoline Scientific™ TWBC Series, Wetherill Park, NSW, Australia). To keep the loggers submerged underwater, the bag of data loggers was housed inside an aluminium mesh cage. Water bath temperature was constant for 50–60 min at four different temperatures: 27°C, 29°C, 31°C and 33°C. Loggers were given at least 15 min to reach a steady state at each new temperature. After this 15-min equilibration period, there was no monotonic change in the recorded temperatures, indicative that each logger had fully responded to the change in temperature. This range of temperatures was selected because they are typical of sea turtle nest temperatures (reviewed in [28]). The true temperature (*X_T_*) of the water bath was measured using a National Institute of Standards and Technology (NIST-traceable) Type K thermocouple (certified accuracy of ± 0.3°C between 0°C and 100°C, ANSI/Z540–1–1994 standard, https://www.nist.gov/) inserted into a portable calibrator device (OMEGA® CL3515R, Omega Engineering Inc., Stamford, CT, USA, https://au.omega.com/). Three temperature measurements recorded from a thermocouple (placed inside the ziplock bag with the loggers) were taken over a 15-min period (5 min apart) at each water bath temperature. The average of these three values was deemed to be *X_T_*. We defined precision as the repeatability of the temperature measurements for each logger at each water bath temperature. We defined accuracy as the difference between the mean logger temperature versus *X_T_* at each water bath temperature.

### (b) Importance of accuracy for sex ratio estimates

For sea turtles, male hatchlings are produced at cooler incubation temperatures and females at warmer temperatures, with a 50 : 50 sex ratio being produced at the pivotal temperature (PT) [27]. We used a well-established and generalized relationship between incubation temperature and hatchling sex ratio for sea turtles [16] to model how differences in data logger temperature accuracy would influence predictions of hatchling sex ratio. For incubation temperatures between 27°C and 33°C, we randomly selected a measurement error (difference from *X_T_*) for each logger type from the observed distribution of accuracy values derived from the water bath trials. For each ‘modelled measured temperature’ (i.e. *X_T_* + measurement error), we calculated the resulting hatchling sex ratio. For each combination of true temperature and logger accuracy, we simulated 200 estimates of hatchling sex ratios. In this way, we determined the difference between the sex ratio estimated from the true temperature versus that estimated from the modelled measured temperature and thereby assessed the implications of logger accuracy. These calculations were run in Minitab v. 8.2 extended.

## 3. Results

### (a) Accuracy and precision of different loggers

**Figure 1.**
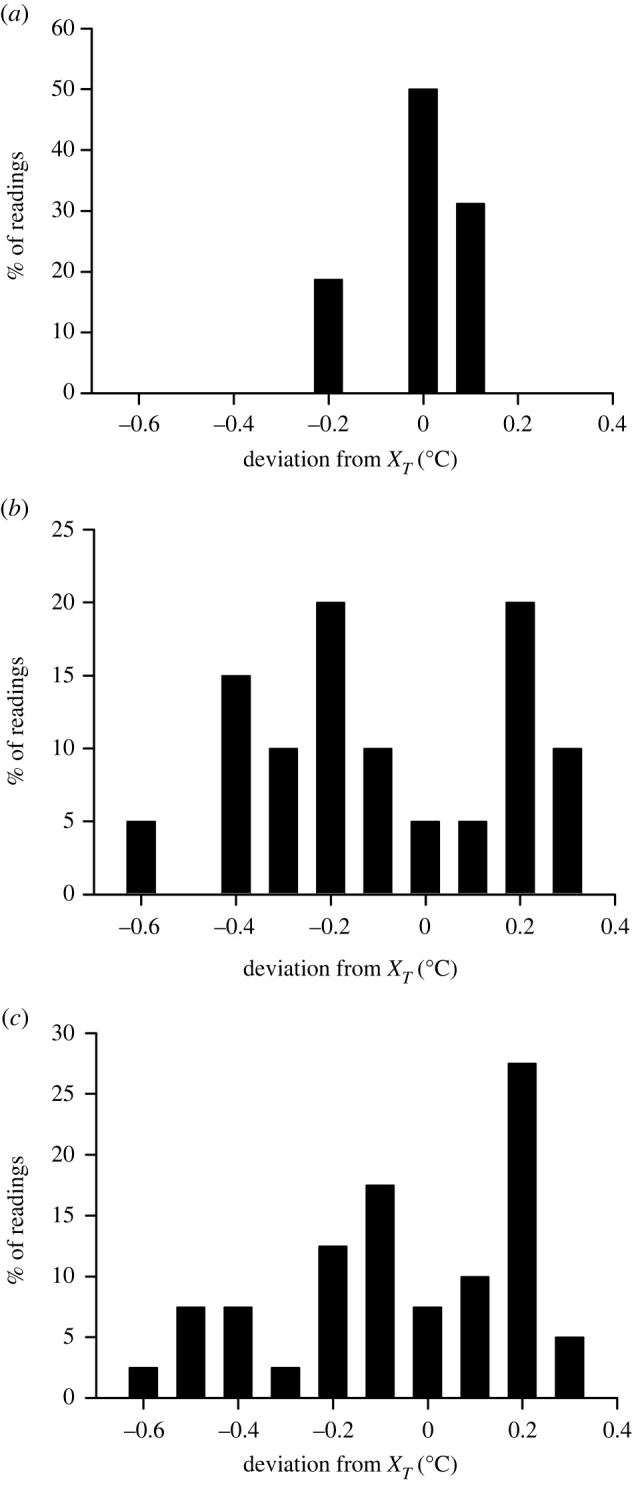
Temperature differences from three brands of data loggers from the certified thermocouple (*X_T_*) at water bath temperatures of 27–33°C. (*a*) TinyTag data loggers, (*b*) HOBO data loggers and (*c*) iButton data loggers.

For all temperature regimes, accuracy was highest in TinyTag loggers (95% of readings within 0.23°C of *X_T_*, mean absolute difference 0.07°C) and lowest in HOBO and iButton loggers with 95% of readings within 0.43°C and 0.47°C of *X_T_*, respectively, and mean absolute differences from *X_T_* being 0.24°C and 0.21°C respectively (figure 1 and table 1). Differences from *X_T_* at each temperature of the water bath for each data logger are presented in the electronic supplementary material, table S2. The precision of each logger, as measured by the mean standard deviation of the repeat measurements at each water bath temperatures were TinyTags = 0.04°C, HOBOs = 0.22°C and iButtons = 0.17°C.

### (b) Implications of logger accuracy

**Figure 2.**
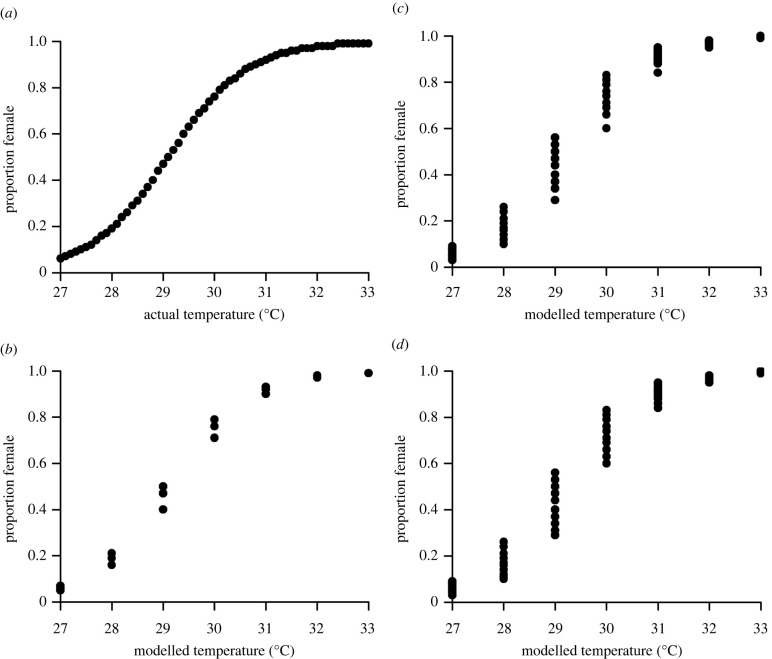
The predicted hatchling sex ratio at different temperatures as a function of temperature logger accuracy. (*a*) The relationship between temperature and sex ratio reported in Hays *et al*. [16]. The relationship between the modelled measured temperatures (*X_T_* + random measurement error from figure 1) and the predicted sex ratio estimates for temperature data theoretically derived from (*b*) TinyTags, (*c*) HOBOs and (*d*) iButtons.

Logger accuracy had relatively little impact on the estimated hatchling sex ratio when *X_T_* was well above or below the PT (figure 2*b–d*). For example, at an *X_T_* of 27°C, highly male-skewed hatchling sex ratios were estimated regardless of the logger accuracy and similarly highly female-skewed hatchling sex ratios were modelled at 32°C and above.

However, closer to the PT, logger accuracy had a greater impact on predicted hatchling sex ratio. For example, at 29°C when the hatchling sex ratio versus temperature curve (figure 2*a*) predicted a sex ratio of 0.47 female, the range of sex ratio estimates for TinyTags was 0.40–0.50 (mean = 0.47, s.d. = 0.04, *n* = 200), for HOBOs 0.29–0.56 (mean = 0.43, s.d. = 0.08, *n* = 200) and for iButtons 0.29–0.56 (mean = 0.45, s.d. = 0.08, *n* = 200). These outcomes suggest that even with the least accurate temperature loggers, logger accuracy will generally not appreciably compromise estimates of sea turtle hatchling sex ratios.

## 4. Discussion

Our findings provide heartening news for the many studies around the world that have used sand temperatures on nesting beaches to estimate hatchling sex ratios (reviewed in [29]). We can report that even the lower accuracy loggers, which are widely used (HOBO and iButton), provide sufficiently reliable estimate of sand temperature when averages from several loggers are used, as is often the case (e.g. [15,30,31]). It should be noted that close to the PT, the estimated sex ratio from a single logger of lower accuracy could still be relatively large. Notwithstanding, in planning their studies ecologists should carefully consider the trade-off between the cost of data loggers and the number of loggers required to test their hypothesis given the logger's accuracy. Therefore, higher logger accuracy means that fewer loggers are needed to determine sand temperature reliably and accurately.

Data loggers have become essential tools in many ecological studies, such as recording animal location, ambient temperature, depth or altitude and assessments of accuracy allow better interpretation of the results from these studies [32,33]. For example, an assessment of location accuracy in movement studies allows an improved assessment of patterns of movement [34]. In some cases, details regarding data logger accuracy supplied by manufacturers are somewhat vague. For example, while the manufacturers of the TinyTag, HOBO and iButton data loggers all report an accuracy of ±0.5°C, it is not clear how this value is calculated. Encouragingly, we have shown that temperature recorded by the three loggers we tested are less than 0.5°C from the *X_T_*. This logger accuracy is sufficient to reliably estimate hatchling sex ratios provided data from individual loggers are not over-interpreted when close to the PT and as long as accuracy is maintained over the life of the logger. Some scientists might have research questions in which a higher accuracy of temperature measurement is critical. In either case investigators should carefully consider and report accuracy, as some have done (e.g. [26,33,35]).

Response time is an additional variable that should be considered when selecting the most appropriate device (e.g. [36]). Despite all loggers having responses of a few minutes in this study, a fast reaction time is seldom important for most sand temperature monitoring studies as the focus is temperature trajectories over days or weeks [15,24]. In some cases, a rapid thermal response time might be important. For example, when assessing the variation in temperature with depth for a diving marine animal, where it is critical that a data logger has a rapid thermal response time [33].

We showed that the data from individual loggers (representing microclimates on a beach) can provide a reliable estimate of hatchling sex ratio from those measured temperatures. The high accuracy we found for all three brands of temperature logger, suggests that recording the natural spatial variations in sand temperature will often be more important than logger accuracy when trying to estimate hatchling sex ratios for an entire beach. It is also known that for loggers deployed above ground and in direct sunlight, there can be large errors arising from the absorption of solar radiation [26]. Furthermore, estimates of hatchling sex ratios derived from sand temperatures will be impacted by several other factors such as the PT, the transitional range in temperatures (TRT) which can vary across populations and species (reviewed in [27]), and length of the thermal sensitive period (TSP) during incubation when sex is determined [35]. Further refinements for assessing accuracy could include the consideration of how accuracy varies across a broad range of measurement. For example, manufacturers of TinyTag TGP-4017 loggers report that accuracy varies across the measurement range of these devices (−40°C to +85°C), which might sometimes be important, for example, the wider range of variations associated with seasonal fluctuations in temperature at high latitudes.

Temperature measurements are integral to a wide range of ecological studies on organisms because ambient temperature influences the metabolic rate of animals and their locomotory performance [37]. For example, the thermal dependence of the metabolic rates of loggerhead turtles (*Caretta caretta*) and green turtles (*Chelonia mydas*) means that their maximum dive durations vary as a function of ambient water temperature [38,39]. Only in rare situations, where temperature measurement must be accurate to less than 0.5°C will very high accuracy data loggers be needed, so most commercially available temperature loggers will likely provide useful data.

In conclusion, we have shown how the accuracy and precision of temperature loggers can be assessed and how the performance of loggers should be considered in terms of the ecological conclusions that are reached from temperature data. Encouragingly, of the three temperature data loggers we examined, which are all widely used in studies around the world, in the context of sea turtle hatchling sex ratios, all should provide reliable and useful temperature data, particularly when data from individual loggers are not over-interpreted.

## Data Availability

The data from this study are provided in table S2 of the electronic supplementary material [40].
